# Utility of Enabling Technologies in Spinal Deformity Surgery: Optimizing Surgical Planning and Intraoperative Execution to Maximize Patient Outcomes

**DOI:** 10.3390/jcm14155377

**Published:** 2025-07-30

**Authors:** Nora C. Kim, Eli Johnson, Christopher DeWald, Nathan Lee, Timothy Y. Wang

**Affiliations:** 1 Department of Neurosurgery, NYU Grossman School of Medicine, New York, NY 10016, USA; 2 Department of Neurosurgery, Rush University Medical Center, Chicago, IL 60612, USA; 3 Department of Neurosurgery, Duke University School of Medicine, Durham, NC 27710, USA; 4 Midwest Orthopaedics at Rush, Chicago, IL 60612, USA

**Keywords:** scoliosis, adult spinal deformity, advances, technology, challenges

## Abstract

The management of adult spinal deformity (ASD) has evolved dramatically over the past century, transitioning from external bracing and in situ fusion to complex, technology-driven surgical interventions. This review traces the historical development of spinal deformity correction and highlights contemporary enabling technologies that are redefining the surgical landscape. Advances in stereoradiographic imaging now allow for precise, low-dose three-dimensional assessment of spinopelvic parameters and segmental bone density, facilitating individualized surgical planning. Robotic assistance and intraoperative navigation improve the accuracy and safety of instrumentation, while patient-specific rods and interbody implants enhance biomechanical conformity and alignment precision. Machine learning and predictive modeling tools have emerged as valuable adjuncts for risk stratification, surgical planning, and outcome forecasting. Minimally invasive deformity correction strategies, including anterior column realignment and circumferential minimally invasive surgery (cMIS), have demonstrated equivalent clinical and radiographic outcomes to traditional open surgery with reduced perioperative morbidity in select patients. Despite these advancements, complications such as proximal junctional kyphosis and failure remain prevalent. Adjunctive strategies—including ligamentous tethering, modified proximal fixation, and vertebral cement augmentation—offer promising preventive potential. Collectively, these innovations signal a paradigm shift toward precision spine surgery, characterized by data-informed decision-making, individualized construct design, and improved patient-centered outcomes in spinal deformity care.

## 1. Introduction

The treatment of spinal deformity has evolved over centuries, shaped by innovations in biomechanics, imaging, and surgical instrumentation. In its earliest forms, deformity management relied on rudimentary mechanical traction and external bracing, with variable outcomes and little understanding of the spine’s three-dimensional complexities. These nonoperative methods dominated until the 20th century, when the limitations of passive correction and high rates of progression in untreated scoliosis spurred a search for more reliable, mechanical means of stabilization [1,2].

The recognition that spinal deformity is inherently three-dimensional, encompassing coronal curvature, sagittal malalignment, and axial vertebral rotation, was a pivotal moment. Pioneers like Dubousset and Stagnara emphasized the need for multiplanar correction, introducing concepts such as the “cone of economy” and the spinal “chain of balance,” which reframed deformity not merely as a structural aberration but as a disruption in global postural alignment [3]. Improvements in imaging, especially the transition from 2D radiographs to 3D stereoradiographic modalities, enabled more accurate characterization of deformity morphology and surgical outcomes [4].

The surgical management of spinal deformities is among the most technically demanding challenges in both pediatric and adult spinal care. Over the past century, advancements in spinal instrumentation, surgical techniques, and biomechanics have transformed the field. Historically, nonoperative strategies—such as external bracing and skeletal traction—dominated treatment paradigms. However, these primitive techniques failed to address deformity correction in cases of even moderate severity. Rigid external orthoses such as the Milwaukee and Boston braces provided coronal control but offered minimal influence on sagittal or axial alignment, and patient compliance was often limited by discomfort and social stigma [5]. Early surgical interventions, including fusion without instrumentation, carried high pseudarthrosis rates and required prolonged immobilization [6].

In the early 20th century, Fritz Lange introduced the usage of internal metallic fixation to stabilize the spine. This represented a hallmark transition towards instrumentation for deformity correction. However, few advancements were made beyond this until the introduction of Harrington rods in the mid-20th century. While revolutionary, Harrington instrumentation was associated with significant limitations, notably iatrogenic flatback deformity and inadequate sagittal control, which inspired the development of more advanced systems [7]. As surgical instrumentation advanced, so too did the ability to execute powerful corrective maneuvers across all three planes, reducing the historical reliance on postoperative bracing and lengthy hospitalizations.

Luque’s sublaminar wiring system in the 1970s introduced segmental fixation that more effectively distributed corrective forces. This was followed in the 1980s by the Cotrel–Dubousset (CD) system, which incorporated contoured rods and transverse connectors to facilitate three-dimensional correction, including axial de-rotation. These developments were foundational for the next major leap: the widespread adoption of pedicle screw fixation.

The development of vertebral and pedicle screw fixation has arguably been the most transformative advancement in spinal deformity correction. The concept dates back to King’s early use of vertebral screws in 1944 and Boucher’s description of transpedicular screw fixation in 1959. However, the turning point came with Roy-Camille’s introduction of a true pedicle screw-plate construct in the 1970s, which laid the biomechanical groundwork for modern posterior spinal instrumentation. Subsequent designs, including the Wiltse and Steffee systems, introduced monaxial and polyaxial screw heads and modular rod connections, increasing the versatility and strength of deformity constructs, particularly in adult spinal deformity (ASD). These innovations significantly reduced dependence on postoperative external immobilization, improved load distribution, and enabled stronger corrective maneuvers across all planes [8].

By the 1990s, pedicle screw constructs became the standard for three-column fixation, facilitating robust deformity correction and higher fusion rates. Minimally invasive adaptations—including thoracoscopic approaches—further improved outcomes in selected patients by reducing surgical morbidity and accelerating recovery [8]. As technologies improved, spinal deformities once thought to be untreatable are now increasingly undergoing surgical intervention. In addition to biomechanical constraints, the increasingly geriatric and iatrogenic spinal deformity population also poses additional medical challenges that must undergo special consideration, which is the focus of a large body of prospective clinical research.

The original pioneers of spinal deformity surgery relied on trial and error as well as the wisdom gained from many years of both successes and failures. The new age of spinal deformity is instead defined by precision-based, data-driven approaches. Technological innovations—such as stereoradiographic imaging, segmental bone density mapping, robotic instrumentation, and artificial intelligence—now permeate every phase of surgical planning and execution. This review critically examines the evolution of deformity correction, with a particular focus on enabling technologies that have redefined the surgical landscape and continue to advance the boundaries of what is surgically possible.

## 2. Modern Technology

### 2.1. Advances in Deformity Imaging

**Figure 1:**
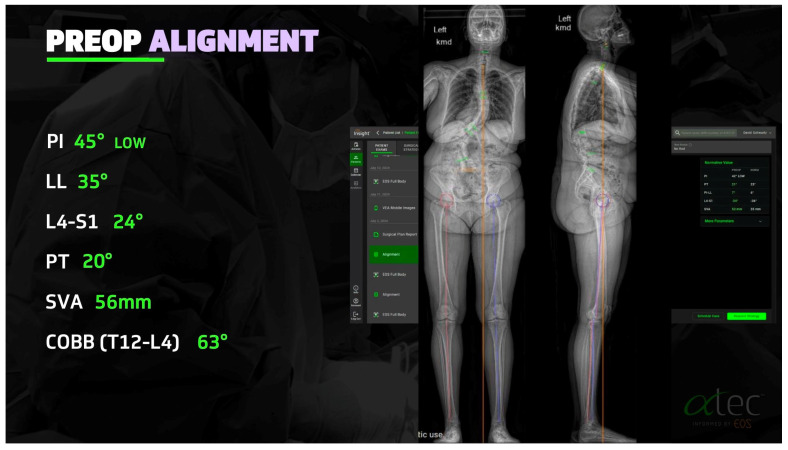
Example of AlphaTec EOS imaging with measurements of spinopelvic alignment.

The shift toward data-centric and individualized deformity correction begins with innovations in imaging and radiographic analytics. Traditional radiographs have given way to low-dose, full-body stereo radiographic imaging systems such as EOS, which allow for upright, biplanar assessment of global spinal alignment in three dimensions (Figure 1). EOS technology facilitates precise measurements of key spinopelvic parameters—including pelvic incidence–lumbar lordosis (PI-LL) mismatch, sagittal vertical axis (SVA), and T1 pelvic angle (TPA)—which are now recognized as critical determinants of postoperative outcomes and long-term sagittal balance [9].

Beyond morphometric analysis, EOS imaging can be utilized to derive segmental vertebral bone density through radiographic grayscale values or DEXA-equivalent metrics [10]. This has enabled a new paradigm in preoperative planning: segment-specific assessment of bone quality to guide implant strategy. Patient-specific interbody implants (PSIs) and fixation systems tailored to local bone density—such as that provided through AlphaTec EOS™ platform or Aurora Spine’s DEXA-C™ technology—represent an evolution toward precision osseointegration. These systems allow surgeons to optimize endplate contact, minimize subsidence risk, and enhance biomechanical stability, particularly in osteopenic patients.

Additionally, the use of radiographic planning software (e.g., Surgimap, Nemaris) has facilitated standardized deformity classification, quantification of deformity flexibility, and simulation of planned correction. This enables objective surgical planning, intraoperative translation of targets, and more reproducible alignment outcomes.

Despite these advancements, limitations to the widespread implementation of advanced imaging technologies persist. EOS systems and other stereo radiographic platforms entail significant capital investment, limiting availability to high-resource centers and introducing disparities in access to precision deformity planning. While EOS imaging is associated with substantially reduced radiation exposure compared to conventional radiography or CT, cumulative exposure remains a concern in patients requiring serial imaging for surgical planning and longitudinal follow-up [11,12]. Furthermore, interpretation of 3D reconstructions and grayscale-derived bone metrics requires specialized training and software infrastructure, which may not be universally available across institutions. As these imaging modalities become more integrated into surgical workflows, efforts toward standardization, cost-containment, and broader accessibility will be essential to ensure equitable benefit across the deformity patient population.

### 2.2. Robotic Assistance and Intraoperative Navigation

Robotic assistance has emerged as a transformative adjunct in spinal deformity surgery, enhancing the accuracy, efficiency, and safety of complex instrumentation procedures. Robotic end effectors and software platforms are also theorized to reduce the surgeon’s cognitive and physical work, which may be particularly beneficial for extended deformity cases. Contemporary robotic platforms—such as Mazor X Stealth Edition (Medtronic, Caesarea, Israel), ExcelsiusGPS (Globus Medical, Audubon, PA, USA), and ROSA Spine (Zimmer Biomet, Montpelliar, France)—integrate preoperative imaging with real-time intraoperative navigation, enabling highly accurate pedicle screw placement even in patients with dysplastic or rotated vertebral anatomy. These systems permit execution of a preoperative plan with sub-millimetric precision and reduce the dependency on fluoroscopy, thereby minimizing cumulative radiation exposure for both patients and surgical teams [13,14,15]. In addition, robot-assisted pedicle screw placement has improved accuracy and optimal placement compared to other modalities [16,17]. When combined with patient-specific implants and real-time neuromonitoring, robotic platforms may also support safer correction in frail or comorbid patients.

In addition to general thoracolumbar instrumentation, robotic guidance has demonstrated particular utility in facilitating accurate and efficient pelvic fixation. Placement of S2-alar-iliac (S2AI) or iliac screws traditionally poses technical challenges, particularly in the context of altered sacropelvic anatomy, long-segment constructs, minimally invasive approaches, or revision surgery. Robotic platforms allow for preoperative planning of pelvic trajectories that are colinear with proximal instrumentation, reducing the need for rod contouring, connectors, or angular transitions at the lumbosacral junction [18,19]. This alignment optimization is especially advantageous in constructs requiring dual pelvic fixation in constructs with existing sacropelvic instrumentation (i.e., isolated sacro–iliac fusion screws or trans-sacral trauma constructs). With robotic planning, entry points and trajectories for double-barrel iliac or S2AI screws can be precisely configured to ensure parallel placement with appropriate medialization, depth, and convergence [18,19]. These capabilities enhance construct stability, improve load distribution, and mitigate hardware-related complications at the distal terminus of long fusion constructs, while concurrently reducing intraoperative fluoroscopy and technical variability.

Despite these advantages, limitations persist. Robotic platforms carry a non-trivial learning curve, increased operative setup time, and high capital investment. Notwithstanding its precision advantages, robotic instrumentation demands a non-trivial learning curve. Early series suggest that 15–30 cases are typically required before screw-placement times, workflow efficiency, and fluoroscopy usage plateau; achieving expert-level proficiency may exceed 50 cases, particularly in complex deformity scenarios where multilevel, multi-planar trajectories are executed [20,21]. The learning phase also extends beyond the primary surgeon: accurate registration, arm draping, and collision-avoidance depend on a well-coordinated operative team, necessitating dedicated in-service training, iterative protocol refinement, and continuous performance auditing to translate robotic accuracy into overall operative efficiency and patient benefit. Moreover, robotic guidance is only as reliable as its imaging and registration fidelity; any mismatch can compromise execution. Moreover, robot-assisted pedicle screw placement can be cost-effective in high-volume centers or when perioperative efficiencies and complication reductions are realized, but the initial capital investment remains a significant consideration [22]. Nevertheless, robotic systems are increasingly positioned to be central to deformity correction workflows.

### 2.3. MIS Deformity

Traditionally, deformity correction was performed via extensive open posterior approaches spanning multiple segments across thoracolumbar spine, multiple interbody implants, and multilevel osteotomies. While effective, these approaches are associated with prolonged operative time, substantial blood loss, extended ICU stays, and higher perioperative morbidity—particularly in the aging and medically complex population that comprises much of the ASD cohort [23]. This also places an enormous economic burden on the healthcare system.

**Figure 2:**
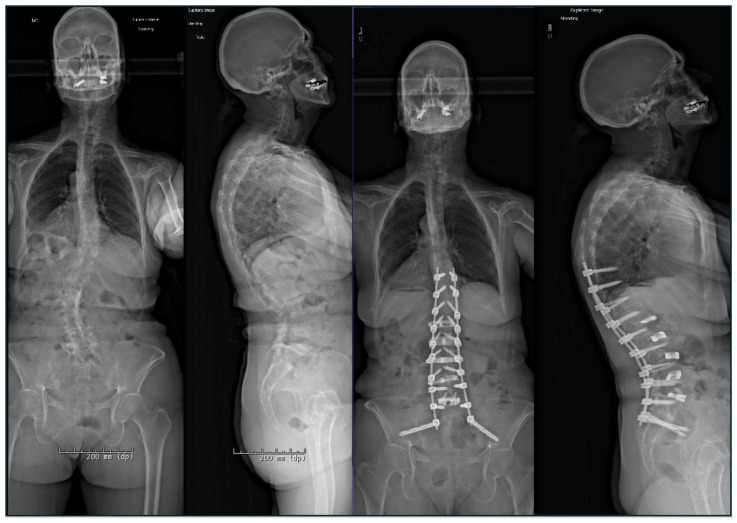
A 71-year-old female with flexible idiopathic lumbosacral coronal deformity presented with worsening back pain, leftward postural lean, and radicular leg pain. She underwent Stage 1: L5-S1 ALIF and Stage 2: L1-5 XLIF from the right (concavity) side. Three days later, she underwent Stage 3: Navigated T10-pelvis fusion through a percutaneous midline skin incision and fascial stab incisions. She did not require any blood product transfusions intraoperatively or postoperatively and did not require an ICU stay at any point. However, her total inpatient time was approximately 11 days.

Contemporary MIS strategies seek to minimize iatrogenic morbidity while achieving comparable alignment and clinical outcomes. The application of hyperlordotic anterior lumbar interbody fusion (ALIF) cages, coupled with lateral lumbar interbody fusion (LLIF or XLIF) open or percutaneous posterior component, represents a growing technique in sagittal and coronal plane realignment (Figure 2). When combined with percutaneous posterior instrumentation, this approach allows for circumferential arthrodesis and restoration of global alignment through smaller incisions, less soft tissue disruption, and oftentimes without the need for extended posterior osteotomies in select cases.

When combined with posterior osteotomy, anterior column realignment (ACR), performed through the lateral transpsoas corridor with hyperlordotic cages and anterior longitudinal ligament (ALL) release enables segmental lordosis restoration up to 30 degrees per level. This technique is particularly powerful when correcting fixed sagittal imbalance in patients who may not tolerate multilevel posterior osteotomies or for those who have rigid lumbosacral deformity from prior surgery. For coronal plane deformities, lateral interbody fusion with strategic implant placement and disc height restoration can provide indirect decompression and reconstitution of coronal alignment with excellent results in appropriately selected patients [24].

Chou et al. demonstrated that circumferential minimally invasive surgery (cMIS) achieved comparable radiographic correction and clinical outcomes to open surgery at 1-year follow-up. While cMIS was associated with significantly reduced blood loss, it involved longer operative times. Rates of achieving minimal clinically important difference and revision surgeries were similar between approaches, supporting cMIS as a viable alternative to open correction in appropriately selected patients [25]. This provides insight into the changing postoperative recovery dynamics [26].

Despite its many advantages, minimally invasive deformity correction is not without limitations. MIS approaches are inherently constrained in their ability to perform extensive posterior column osteotomies, limiting their applicability in patients with rigid, fixed deformities or severe sagittal imbalance that exceeds the correction potential of interbody work alone. Several meta-analyses and systematic reviews demonstrate that while MIS techniques are associated with less intraoperative blood loss and fewer serious postoperative complications, open techniques showed greater improvement in lumbar lordosis and pelvic tilt angle [27,28]. Visualization through tubular or lateral corridors may be suboptimal, particularly in patients with prior surgery, distorted anatomy, or vascular anomalies. Additionally, MIS techniques often require staged procedures, which can prolong total hospitalization and necessitate additional anesthesia exposures. Operative time may also be prolonged in early adoption phases due to limited working corridors and a steep technical learning curve. Furthermore, while early data support comparable alignment outcomes and reduced perioperative morbidity, long-term durability and fusion rates—particularly in patients with poor bone quality—remain under investigation [29,30]. Cost-effectiveness is another consideration, as specialized implants, navigation technologies, and prolonged OR time may offset the savings associated with reduced ICU utilization or length of stay. As such, patient selection, surgeon experience, and institutional infrastructure remain critical determinants of MIS deformity success [31].

### 2.4. Preoperative Planning and Predictive Modeling

Preoperative planning in ASD surgery has evolved from conventional templating to a data-integrated, patient-specific strategy. Advances in imaging, clinical stratification tools, and predictive analytics now allow for more precise operative planning and risk mitigation.

Advanced imaging modalities such as EOS enable low-dose, three-dimensional, upright evaluations of spinal alignment, facilitating accurate assessment of key spinopelvic parameters, including PI-LL mismatch and sagittal vertical axis. These quantitative markers form the foundation of modern surgical decision-making in ASD [9].

Frailty indices and validated comorbidity scoring systems have been developed to predict perioperative risk in complex deformity surgery [32]. For example, the Adult Spinal Deformity Frailty Index (ASD-FI) integrates clinical and functional variables to stratify patients according to the likelihood of postoperative complications, offering a framework for surgical candidacy assessment and perioperative optimization.

**Figure 3:**
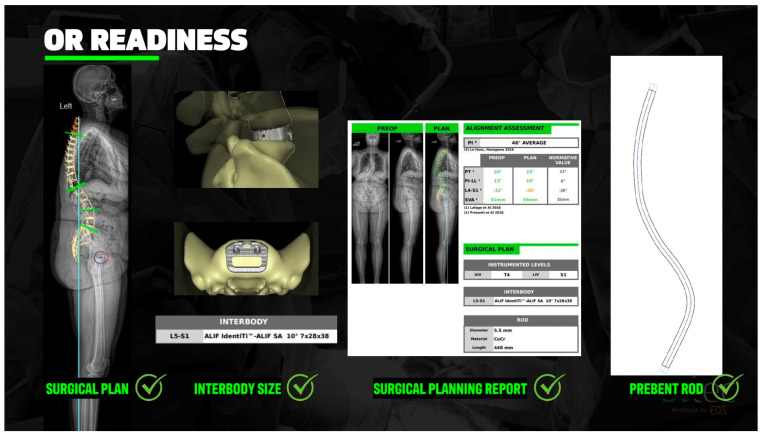
Example of predictive modeling based on preoperative imaging and desired alignment goals. Predictive modeling can show anticipated surgical alignment outcomes based on level—specific intervention. Software shown is provided by AlphaTec EOS.

In parallel, predictive modeling using machine learning algorithms has emerged as a powerful tool for forecasting complications such as proximal junctional kyphosis, pseudarthrosis, extended length of stay, and readmissions (Figure 3). These models leverage high-dimensional clinical and radiographic datasets to refine prognostication and tailor perioperative strategies [32,33]. Artificial intelligence applications are also under active development to automate radiographic analysis and simulate corrective outcomes. Recent work has demonstrated the potential for AI-based platforms to predict spinopelvic parameters and assist in planning osteotomy levels and alignment goals, streamlining both preoperative modeling and intraoperative execution [32].

### 2.5. Machine Learning and Surgeon-Specific Predictive Modeling

Machine learning (ML) and artificial intelligence (AI) are rapidly transforming the landscape of spinal deformity surgery by offering tools capable of processing complex, high-dimensional data to inform risk prediction, surgical planning, and postoperative outcome forecasting. Unlike conventional regression-based models, ML techniques can identify nonlinear interactions among dozens or hundreds of clinical and radiographic variables, making them uniquely suited for applications in heterogeneous populations such as ASD patients [34,35].

Early studies applied ML algorithms—particularly artificial neural networks (ANN), support vector machines, and decision trees—to predict postoperative complications including wound infections, cardiac events, and venous thromboembolism. For example, Kim et al. demonstrated that ANN-based models significantly outperformed traditional ASA scores and logistic regression in predicting major complications after ASD surgery, with improved accuracy across nearly all complication categories [36]. Similarly, Wang et al. introduced a LightGBM model trained on a multicenter cohort that predicted “ideal surgical outcome”—defined as achieving the minimal clinically important difference in patient-reported outcomes without any complications—with an area under the curve (AUC) of 0.888 [37].

As modeling techniques have matured, more sophisticated inputs have been incorporated into ML pipelines. Recent efforts have leveraged deep learning architectures, including multilayer perceptrons and convolutional neural networks, to include radiographic measurements, muscle cross-sectional area, and even lab values in predictive models. Schonfeld et al. used deep learning with standard lab and operative variables to predict revision risk in cervical spine surgery with strong discriminative ability (AUC 0.833), highlighting the potential for integrating routinely collected clinical data into ML frameworks [38].

**Figure 4:**
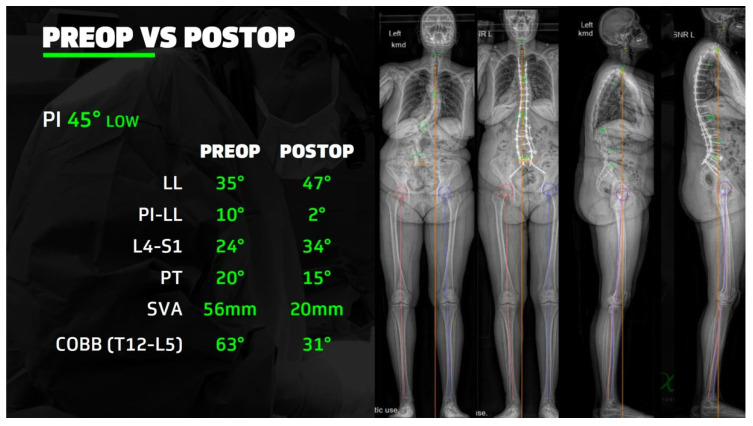
Comparison of preoperative and postoperative outcomes is returned to the machine learning algorithm to provide surgeon-specific learning that then improves the accuracy of future preoperative modeling.

Crucially, machine learning’s power is no longer limited to generalized population-level models. A growing body of work is exploring surgeon-specific and institution-specific predictive modeling (Figure 4). These approaches account for variations in individual surgical technique, institutional protocols, and equipment—variables that traditional models often ignore. In these personalized models, ML learns from a surgeon’s own historical outcomes and can offer feedback for continuous improvement, case selection, or alignment goal planning. Although these tools are in their infancy, they present a compelling vision of iterative, self-improving surgical decision support systems [39].

Despite these advancements, limitations remain. Many existing models suffer from a lack of external validation, poor generalizability across patient populations, and dependence on retrospective, single-institution datasets [33]. Furthermore, there are challenges in clinical adoption, including interpretability (i.e., “black box” models), regulatory oversight, and integration into electronic health records or surgical planning software. Efforts to enhance model explainability—such as the use of Shapley additive explanation (SHAP) values and other forms of interpretable AI—are underway to bridge the gap between predictive performance and clinical usability [40].

In summary, ML applications in spinal deformity surgery have evolved from retrospective proof-of-concept studies to powerful, increasingly interpretable clinical decision support tools. As data sources become richer and more standardized, surgeon-specific predictive analytics may soon play a central role in personalizing care and optimizing outcomes in deformity surgery.

### 2.6. Custom Patient-Specific Interbody Implants

**Figure 5:**
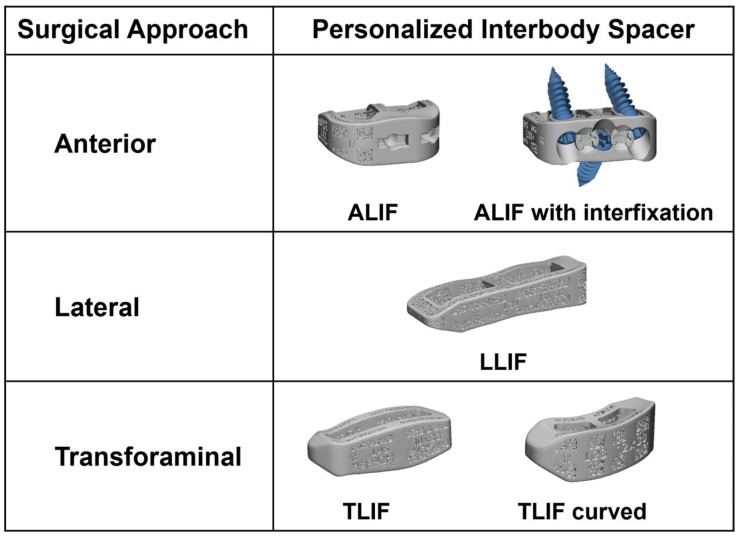
Patient-specific interbody implant types. Figure from Kent et al. and reused under the Creative Commons Licensing Agreement CC BY-NC-ND [44].

Custom, patient-specific interbody (PSI) cages represent a major advancement in spinal reconstruction technology, particularly in the treatment of complex spinal deformity and revision cases (Figure 5). Leveraging high-resolution imaging, computer-aided design (CAD), and additive manufacturing, these implants are engineered to match a patient’s unique endplate anatomy. Compared to standard, off-the-shelf (OTS) cages, PSIs offer improved endplate conformity, optimized contact area, and customizable lordotic or kyphotic angles that align with the surgical correction strategy [41,42].

Traditionally, interbody cages have been limited by the need to accommodate a wide range of anatomies with a narrow range of implant geometries. As a result, surgeons often compromise on fit, which may result in reduced endplate coverage, uneven stress distribution, and increased risk of cage migration or subsidence. Patient-specific cages mitigate these issues by tailoring implant shape, footprint, and trajectory to the recipient anatomy using CT or MRI data. This allows for preoperative simulation of cage placement and correction angles, enhancing both planning and intraoperative confidence [43].

Biomechanical data increasingly support the use of PSIs in achieving superior construct stability. Fernandes et al. conducted a cadaveric comparison between 3D-printed patient-specific cages and commercially available titanium and PEEK devices. Their findings demonstrated a 64% higher peak failure force and a 59% increase in construct stiffness with PSI designs, indicating a significantly lower risk of subsidence under physiologic loads [45]. These mechanical advantages may translate into greater construct durability and fewer alignment losses over time, especially in osteopenic or dysmorphic bone.

Clinical applications of PSIs have been most extensively reported in complex deformity correction, tumor reconstruction, and revision surgery. In a systematic review of 17 studies involving 35 patients, Wallace et al. found that PSI use was associated with high fusion rates and favorable clinical outcomes. Only one revision was required due to symptomatic subsidence, and no cases of implant migration, loosening, or pseudarthrosis were reported [42]. Notably, 11 cases exhibited radiographic subsidence greater than 3 mm, though most were asymptomatic and did not require intervention, suggesting that PSI-related subsidence may be less clinically consequential when implants maintain global stability [43].

Further expanding on clinical efficacy, Laynes and Kleck reviewed multiple PSI case series demonstrating their utility in achieving target spinal alignment. The authors emphasize the value of integrating patient-specific planning software, which allows surgeons to design interbody cages and rods in conjunction with one another, enhancing alignment correction in both coronal and sagittal planes. Particularly in long-segment fusion and sagittal realignment cases, this integrated planning workflow enables the generation of pre-contoured rods and interbodies that reflect the exact angular and spatial requirements for deformity correction [42].

Material science and design features of PSIs also contribute to their potential advantages. 3D-printed titanium alloys, commonly used in PSIs, feature porous surface architectures that enhance osteointegration compared to solid PEEK implants [46]. These materials can be fabricated using techniques such as electron beam melting or selective laser sintering, which allow the incorporation of lattice structures that mimic cancellous bone architecture and promote vascular ingrowth [47].

Despite their promise, barriers to widespread adoption remain. High production costs, longer lead times, and the need for thin-slice CT imaging and design infrastructure can limit access, particularly in resource-constrained settings. Additionally, templated interbody implants have limited flexibility in adapting to intraoperative changes such as endplate violation or access limitations during lateral and anterior approaches. Moreover, the lack of large-scale prospective comparative trials restricts definitive conclusions about long-term efficacy. As highlighted in a 2024 review by Iqbal et al., technological limitations in 3D printing resolution, segmentation accuracy, and regulatory guidance continue to challenge broader implementation [46].

Nevertheless, as 3D printing technologies evolve and planning platforms integrate more seamlessly with surgical workflows, patient-specific interbody cages are expected to play an increasingly central role in complex spinal deformity correction. Their ability to marry mechanical performance with individualized anatomic fit positions them as a cornerstone in the emerging era of precision spinal reconstruction.

### 2.7. Custom Patient-Specific Rods

The integration of patient-specific rods (PSRs) into ASD surgery marks a pivotal advance in individualized alignment correction. Traditional rod contouring relies on intraoperative manual bending, which introduces variability and increases the risk of under- or over-correction, particularly in complex, long-segment constructs. PSRs are designed preoperatively based on individualized surgical alignment plans and manufactured to match target curvatures with high precision, thereby enhancing the execution fidelity of corrective maneuvers and improving the reproducibility of alignment outcomes [48,49].

**Figure 6:**
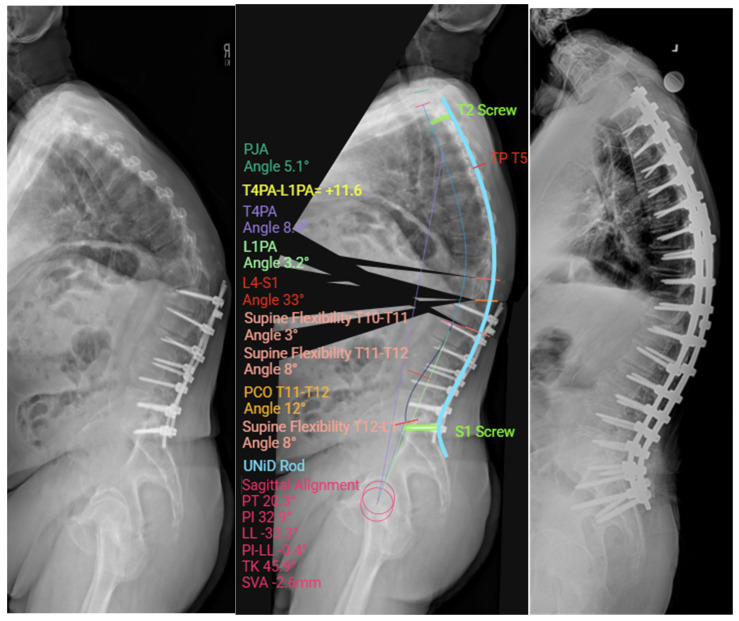
Case example an adult spinal deformity patient with proximal junctional kyphosis after a prior T12-S1 fusion. Medtronic preoperative planning software was used to assist with spinopelvic parameter measurements. This data was incorporated into UNiD rod planning to produce a prefabricated rod for optimum surgical result. The subsequent postoperative alignment accurately reflects the preoperatively planned goal.

PSRs are typically developed using advanced imaging data (e.g., EOS or CT), computer-aided design, and planning software such as UNiD™ Adaptive Spine Intelligence v4.0 (Figure 6). These systems generate a deformity correction plan based on goal spinopelvic parameters—such as PI-LL mismatch, SVA, and pelvic tilt (PT)—which are then used to pre-contour rods accordingly [50,51]. This transition from intraoperative improvisation to preoperative optimization has led to significant improvements in radiographic outcomes in ASD and significantly reduced cognitive load for a critical portion of deformity surgery.

A 2024 systematic review by Picton et al. aggregated data from 304 ASD patients across seven studies and found consistent improvement in SVA and PI-LL following PSR-guided surgeries, with some studies demonstrating a strong correlation between preoperative plans and postoperative results [48]. Likewise, Bautista et al. noted that PSRs contributed to enhanced radiographic alignment in both adolescent and adult populations, while also reducing operative time and blood loss by eliminating the need for intraoperative rod shaping [51].

Multiple prospective studies support these findings. In a cohort of 86 patients, Prost et al. demonstrated that PSR implementation significantly improved sagittal alignment parameters at one-year follow-up, including PI-LL mismatch and SVA. Additionally, fewer patients required postoperative revisions for mechanical complications compared to historical controls [52]. Similarly, Faulks et al. reported that PSRs led to decreased rates of proximal junctional failure (PJF), improved patient-reported outcome measures (PROMs), and a strong correlation between planned and achieved alignment targets over 24 months of follow-up [53].

Comparative analyses further validate the benefits of PSRs. Nasto et al. conducted a matched cohort study comparing PSRs to traditional rods and found that although both groups experienced improvements, only the PSR group reliably achieved preoperative sagittal correction targets such as global tilt and PI-LL. Clinical outcomes and complication rates were similar between the groups, indicating that PSRs may improve alignment precision without added risk [54].

Importantly, the use of PSRs may reduce intraoperative error and surgeon fatigue. Solla et al. highlighted that patients receiving PSRs were 2.6 times more likely to achieve optimal PI-LL alignment compared to those with traditional instrumentation, a finding attributed to improved execution of preoperative plans and reduced variability in rod contouring [55].

In addition to pre-manufactured patient-specific rods (PSRs), the Bendini^®^ system (NuVasive) offers an intraoperative solution that utilizes digital navigation and real-time pedicle screw head alignment to contour rods with greater precision than traditional manual techniques. Unlike freehand contouring with French benders, Bendini generates a rod trajectory based on tulip positions and applies a series of notched bends using a proprietary bender, which may enhance reproducibility but has also raised concerns regarding localized weakening at the bend points. A 2023 study by Takeuchi et al. evaluated the accuracy of intraoperative coronal alignment using the Bendini system in adult spinal deformity surgeries. The study found a strong correlation between intraoperative measurements and postoperative radiographs, suggesting that Bendini can effectively reduce reliance on intraoperative radiography and fluoroscopy, thereby minimizing radiation exposure [56].

An emerging innovation in spinal instrumentation is the use of Molybdenum-Rhenium (MoRe^®^) rods, a proprietary superalloy designed to offer superior mechanical and biological performance in spinal deformity constructs. Compared to traditional cobalt-chromium and titanium rods, MoRe^®^ demonstrates significantly higher yield strength and fatigue resistance, which may reduce the risk of rod fracture—especially in high-stress constructs such as those involving pedicle subtraction osteotomies (PSOs). Prior multicenter analyses have shown rod fracture rates of up to 22% in such cases, emphasizing the need for more durable materials [57]. Beyond mechanical durability, MoRe^®^ exhibits promising biological properties. In a recent in vitro study, Molz et al. compared ion release profiles of MoRe^®^ rods to conventional alloys and found that MoRe^®^ produced significantly lower concentrations of cobalt, nickel, and chromium ions—elements known to induce inflammatory responses and impair osteointegration. These results suggest that MoRe^®^ may reduce the adverse biological environment associated with conventional metallic implants and potentially improve long-term fusion outcomes [58].

Despite these advantages, limitations remain. Prost et al. noted that even with PSRs, a subset of patients remained malaligned postoperatively, and radiographic overcorrection may persist in some cases [52]. Additionally, factors such as software planning accuracy, implant delivery logistics, and cost must be considered. As with PSIs, wider adoption will require streamlined integration into clinical workflows and demonstration of long-term benefit through randomized controlled trials.

Nevertheless, the convergence of planning software, machine learning algorithms, and robotic rod fabrication represents a promising frontier in deformity correction. As data accumulates and surgical planning becomes more sophisticated, patient-specific rods are expected to become a core component of precision spine surgery, particularly in long-segment deformity reconstructions and revision cases.

### 2.8. PJK Prevention: Tethers, Hooks, Kyphoplasty

Proximal junctional kyphosis (PJK) and its more severe counterpart, proximal junctional failure (PJF), remain among the most common and debilitating complications following long-segment ASD surgery. While PJK is typically radiographic, PJF represents a structural failure often necessitating revision surgery. The incidence of PJK has been reported to range from 20 to 40%, with PJF affecting up to 20% of patients depending on surgical factors, bone quality, and the length of fusion [55]. Despite alignment strategies aimed at achieving ideal sagittal correction—such as age-adjusted PI–LL targets—PJK and PJF continue to occur in a significant subset of patients, prompting the development of preventative surgical techniques [59].

#### 2.8.1. Tethering Techniques

Posterior ligamentous tethering has gained traction as a biologically friendly adjunct to prevent PJK by reinforcing the posterior tension band. Techniques vary from polyester or polyethylene tethers secured to spinous processes or laminae, to synthetic bands affixed via screws or rods. A pilotstudy by Buellet al. demonstrated that the group with spinal tethering through the spinous processes of UIV + 1 and UIV − 1 significantly reduced the rate of PJK compared to the group with no tethering [60]. These findings are consistent with earlier pilot studies suggesting that tethers offload biomechanical stress from the proximal junction while preserving a degree of flexibility that rigid instrumentation lacks.

In addition, a finite element study by Buell et al. evaluated 15 different posterior tethering configurations and demonstrated that UIV + 2 loop or weave tethers most effectively reduced the junctional range of motion and intradiscal pressure. The study also highlighted an optimal tether preload tension (approximately 100 N), above which biomechanical benefits diminished. These findings support the concept that tether configuration and tension play key roles in preventing PJK [61]. Another finite element analysis by Bess et al. similarly demonstrated that posterior polyester tethers anchored cranially to the upper instrumented vertebra (UIV) significantly reduced intervertebral range of motion, intradiscal pressure, and pedicle screw forces compared to constructs with pedicle screws or transverse process hooks. The study supported the hypothesis that a multilevel posterior tether configuration (e.g., UIV + 3) provided a smoother biomechanical transition from instrumented to mobile spine segments, potentially reducing the mechanical risk for PJK [62].

However, tethering is not without limitations. Though revision surgery for PJK was reduced in some tethered groups, the reduction did not always reach statistical significance. This discrepancy may be attributed to differences in tether placement techniques, variability in PJK definitions, or the timing of radiographic assessments [60,63].

#### 2.8.2. Proximal Anchor Modifications: Hooks vs. Screws

The choice of proximal fixation method at the upper instrumented vertebra (UIV) is another important consideration. Pedicle screws, while providing robust anchorage, may concentrate stress at the screw–bone interface, increasing the risk of vertebral fracture. In contrast, transverse process hooks (TPHs) distribute stress over a broader area and may better preserve posterior soft tissue attachments.

Matsumura et al. compared TPHs to pedicle screws in 39 ASD patients and found a lower overall incidence of PJK in the hook group (17.6% vs. 27.3%). Additionally, the degree of kyphotic change was significantly smaller in the hook cohort (5.0° vs. 19.0°, *p* = 0.04), suggesting a potential protective biomechanical effect [64]. Similarly, Park et al. found in a 2024 multivariate regression analysis that the absence of TPHs was associated with a 5.6-fold increased risk of PJF, even in patients who had achieved ideal age-adjusted sagittal alignment [59].

While TPHs may reduce mechanical stress and mitigate junctional failure risk, they may also offer less rigid fixation, which could influence construct stiffness and the ability to maintain long-term alignment. Consequently, the decision between hooks and screws must be individualized, often depending on bone quality, junctional flexibility, and surgeon preference.

#### 2.8.3. Vertebral Cement Augmentation (e.g., Kyphoplasty, Vertebroplasty)

**Figure 7:**
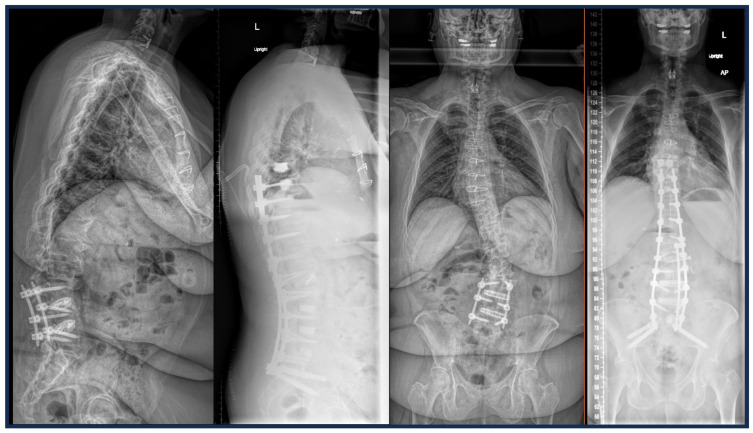
Case example of a patient with prior L3-5 lateral lumbar interbody fusion who then underwent L2-3 lateral lumbar fusion with anterior column release, L5-S1 anterior lumbar interbody fusion, and extension of prior fusion caudally to the pelvis and cranially to T9. Cement augmentation was used at the UIV and vertebroplasty performed at UIV + 1.

Cement augmentation of the UIV and UIV + 1 vertebrae has been explored as a method to reinforce vertebral strength and reduce the incidence of junctional failure, particularly in osteoporotic patients (Figure 7). Theologis and Burch described a technique involving prophylactic kyphoplasty at the two levels above the construct, reporting no cases of junctional fractures in their series, and attributing this to biomechanical load distribution [65].

Raman et al. conducted a prospective 5-year follow-up study of 39 ASD patients who underwent prophylactic vertebroplasty at UIV and UIV + 1 and observed a 5.1% incidence of PJF, which is markedly lower than reported averages. While this study did not demonstrate a significant reduction in long-term PJK incidence, it did support the protective effect of vertebroplasty against early catastrophic failure [66]. Importantly, this technique did not increase perioperative complications or negatively impact functional outcomes.

However, other reports have questioned the longevity of cement-based strategies, citing limitations such as altered adjacent segment biomechanics and uncertainty regarding long-term efficacy [55]. Additionally, when cement augmentation is used during revision surgery for PJF, it may reduce recurrence risk; patients without cement augmentation at UIV during revision were nearly 5.5 times more likely to experience recurrent PJF in a 2024 analysis of 60 revision cases [63].

## 3. Modern Medical Management

### 3.1. Bone Health Optimization

Successful spinal deformity correction relies not only on parameters that can be modified with modern technologies, but also on the underlying innate biology of spinal fusion and instrumentation fixation. Poor bone quality remains a major contributor to implant failure, pseudarthrosis, junctional failure, and subsidence—especially in older adults undergoing long-segment arthrodesis. This is a prevalent issue as osteoporosis is the most common metabolic bone disease in the United States [67]. As such, comprehensive bone health optimization has emerged as a central pillar in deformity surgery, warranting a systematic, multidisciplinary approach.

#### 3.1.1. Preoperative Evaluation

The preoperative period represents a critical window for assessing skeletal integrity and fracture risk. In fact, the Congress of Neurological Surgeons (CNS) released guidelines for preoperative osteoporosis assessment in 2021 [68]. Dual-energy X-ray absorptiometry (DEXA) remains the standard for evaluating areal bone mineral density (BMD) and T-score classification, with thresholds of T < −1.0 for osteopenia and T < −2.5 for osteoporosis per WHO criteria. However, DEXA alone may underestimate regional deficits—particularly in the spine, where degenerative changes can artifactually elevate BMD. Advanced imaging modalities, including high-resolution CT-derived Hounsfield unit (HU) mapping, offer segmental assessments of bone quality at planned screw trajectories and interbody levels. Vertebral HU values < 110 have been associated with increased risk of pedicle screw loosening and cage subsidence [69]. The CNS guidelines demonstrate that preoperative CT HU values less than 97.9 are associated with increased risk of instrumentation failure.

Laboratory evaluation should include serum 25-hydroxyvitamin D, calcium, phosphorus, parathyroid hormone (PTH), and markers of bone turnover (e.g., P1NP, CTX). These data help delineate underlying metabolic bone disorders or secondary contributors to skeletal fragility, such as renal osteodystrophy, vitamin D deficiency, or hyperparathyroidism [70]. Risk stratification tools such as FRAX may also be useful in surgical counseling and intervention planning [71].

#### 3.1.2. Medical Intervention

Pharmacologic management should be initiated preoperatively in at-risk patients to optimize osseous metabolism. The American Society for Bone and Mineral Research (ASBMR) recommends initiating pharmacologic therapy for patients with osteoporosis or high fracture risk who are undergoing spine reconstruction, with anabolic agents such as teriparatide or romosozumab considered first-line in appropriately selected individuals. These agents have demonstrated superior efficacy in promoting spinal fusion and reducing implant-related complications compared to antiresorptives alone. In particular, romosozumab’s dual anabolic and antiresorptive mechanism offers a compelling option for enhancing perioperative osseous integration in high-risk patients, and may be preferable in the setting of severe osteoporosis or delayed fusion potential, particularly when T-score is <−2.5 or history of fragility fracture is present [72,73,74,75]. Antiresorptive agents, such as bisphosphonates, remain a reasonable alternative for patients who cannot receive anabolic therapy, as they still confer benefits in reducing osteoporosis-related complications and improving patient-reported outcomes compared to no treatment. The CNS guidelines recommend teriparatide as first-line therapy in this setting, with antiresorptives as secondary options [68].

Pharmacotherapy should ideally be initiated 4–6 weeks preoperatively and continued through the early postoperative period. In patients already on long-term bisphosphonate therapy, a drug holiday or transition to an anabolic agent may be warranted to avoid oversuppression of bone turnover. Multidisciplinary coordination with endocrinology or bone metabolism specialists is advised in complex or refractory cases [76,77].

#### 3.1.3. Postoperative Management

Postoperatively, ongoing bone health monitoring is essential to ensure maintenance of implant stability, fusion progression, and long-term construct durability. Serial DEXA scanning with repeat laboratory assessment of vitamin D and bone turnover markers as clinically indicated are recommended with pharmacologic therapy in conjunction.

## 4. Conclusions

The trajectory of spinal deformity surgery is poised to accelerate along several converging technological and clinical fronts. Integration of real-time intraoperative analytics with adaptive robotics may usher in an era of responsive deformity correction, wherein surgical plans evolve intraoperatively based on patient-specific biomechanical feedback. Rather than executing fixed trajectories, robotic platforms may soon allow for dynamic correction strategies that adapt in real time to intraoperative changes in alignment, soft tissue tension, or neuromonitoring signals—thereby enhancing precision while reducing reliance on extensive preoperative assumptions. This would represent a shift from static to interactive deformity correction, with the potential to improve outcomes and reduce reoperation rates.

Surgeon- and institution-specific machine learning (ML) models will further transform decision-making by embedding predictive analytics directly into surgical workflows. These models—trained on local historical outcomes and refined over time—will enable granular risk stratification, personalized alignment goals, and construct optimization tailored to each surgeon’s technique and patient population. As a result, ML may transition from a retrospective analytic tool to a prospective surgical co-pilot, capable of enhancing consistency, reducing complications, and guiding postoperative management in real time.

Expanded use of patient-specific implants, including pre-contoured rods, interbodies, and customizable construct ecosystems, will likely redefine mechanical integration at every level of the fusion. These implants allow for precise translation of preoperative alignment targets into the operative field, reducing human variability in rod bending, improving endplate conformity, and enhancing long-term construct stability. As additive manufacturing technologies continue to evolve, the reduced lead times and broader accessibility of custom devices will make precision spinal reconstruction increasingly feasible—even in high-volume or community settings.

Efforts to prevent junctional failure will also become more targeted and data-driven. Rather than applying blanket strategies such as tethers or cement augmentation, future paradigms may incorporate predictive models that forecast junctional stress and recommend prophylactic interventions based on individual risk profiles, bone quality, and construct biomechanics. This could allow for truly preventative structural tailoring at the most failure-prone transition zones.

Finally, the success of this ecosystem will depend on the interoperability of imaging platforms, navigation systems, planning software, and electronic health records. Seamless integration across these domains will be essential to realize the full potential of AI-enabled, digitally assisted deformity surgery. Cloud-based surgical planning, automatic data capture, and real-time intraoperative feedback loops may ultimately produce self-improving systems that enhance surgical safety, efficiency, and long-term durability.

Taken together, these modalities represent more than incremental improvements—they mark a paradigm shift toward biologically responsive, mechanically precise, and data-guided deformity correction. The future will not be defined by any single innovation, but by the synergistic convergence of technologies that empower spine surgeons to deliver safer, more durable, and more personalized solutions to one of the most complex challenges in modern surgery.
